# Unique Case of Cardiogenic Shock in the Setting of Cumulative Calcium Channel Blocker Toxicity

**DOI:** 10.7759/cureus.36762

**Published:** 2023-03-27

**Authors:** Muhammad A Afzal, Sacide S Ozgur, Ariana R Tagliaferri, Lefika Bathobakae, Fayez Shamoon

**Affiliations:** 1 Internal Medicine, St. Joseph's Regional Medical Center, Paterson, USA; 2 Cardiology, St. Joseph's Health, Paterson, USA

**Keywords:** atrial flutter, hypertensive emergency, cumulative ccb toxicity, cardiogenic shock, calcium channel blocker toxicity

## Abstract

Calcium channel blockers (CCBs) are the most prescribed medications in clinical practice. These drugs treat many conditions, including migraine headaches, vasospasms, abnormal heart rhythms, and hypertension. This widespread use, however, has also been linked with the increased incidence of CCB toxicity cases. CCB toxicity may be from accidental ingestion or iatrogenic. Patients may show signs of cardiovascular toxicity such as hypotension, bradyarrhythmia, coma, or even death. The treatment includes discontinuing the offending medication, securing the airway, and raising blood pressure.

Herein, we report a rare case of a 40-year-old male with a history of uncontrolled hypertension and advanced kidney disease who experienced iatrogenic cumulative calcium channel blocker toxicity while switching CCB classes due to a hypertensive emergency with concomitant atrial flutter. Although uncommon in clinical practice, iatrogenic CCB toxicity is possible and equally lethal. Clinicians must be cautious when initiating these drugs, switching between oral and intravenous formulations, or switching from one class to another to avoid overdoses.

## Introduction

Calcium channel blockers (CCBs) are a class of medications prescribed for a plethora of conditions such as hypertension, headaches, various tachycardias, and vasospastic or Raynaud’s disease [1]. CCBs treat these diseases by relaxing vascular smooth muscles via inhibition of L-type voltage-gated calcium channels, which can also cause negative chronotropy and dromotropy [1]. There are two subclasses of CCBs, which are immediate and extended-release formulations [1,2].

Each CCB subclass has a different affinity for myocardium and vascular smooth muscle [2]. The first subclass of CCBs is dihydropyridines which include medications such as amlodipine, nifedipine, felodipine, and nicardipine [1].

Dihydropyridines have strong vasodilator effects, especially on peripheral vascular smooth muscle [1,2]. However, they are less influential on cardiac pacemakers and myocardial contractility [2]. The second subclass of CCBs is non-dihydropyridines, which include medications such as verapamil and diltiazem [1]. Verapamil is a potent vasodilator and suppressor of cardiac contractility, sinoatrial (SA) nodal automaticity, and atrioventricular (AV) nodal conduction [2]. Diltiazem's effects are similar to verapamil, with less vasodilation and more potency on chronotropy [2].

The increased use of these medications and widespread indications have led to both iatrogenic poisoning and intentional or accidental overdoses [1,3]. As per the American Association of Poison Control Centers' National Poison Data System (NPDS) 2020 Annual Report, 6132 CCBs single exposures were reported in the United States, which led to 101 major adverse outcomes and 45 cases of death [1,3]. Data from 2011 show that CCB toxicity was the cause of 78 deaths after 11,764 exposures [4].

Although accidental ingestion was the cause of exposure in 84% of patients, clinicians should be aware of the consequences of iatrogenic toxicity and be mindful of switching formulations and subclasses when prescribing [1].

## Case presentation

A 40-year-old male with a past medical history of uncontrolled hypertension, advanced chronic kidney disease (CKD) not on hemodialysis, mild intermittent asthma, and non-compliance presented to the emergency department (ED) complaining of dyspnea on exertion for one week that progressively worsened to dyspnea at rest. He denied any complaints of fevers, chills, cough, chest discomfort, orthopnea, paroxysmal nocturnal dyspnea, or leg swelling. He also denied any recent travel or sick contacts.

**Figure 1:**
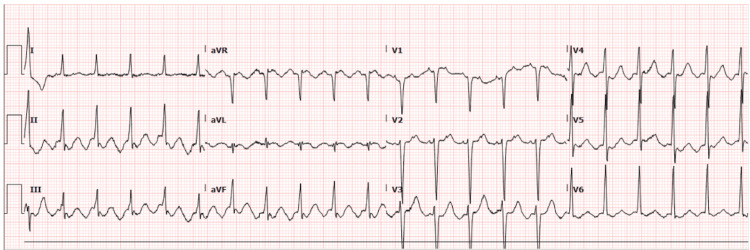
Initial electrocardiogram

Initial vital signs at the time of presentation were as follows: blood pressure, 210/140 mmHg; heart rate, 130 beats per minute (bpm), respiratory rate, 24 breaths/minute; temperature, 36.5 Celsius; and BMI, 30.4. His physical examination was significant for tachycardia with an irregular rhythm; his lungs were clear to auscultation, and no jugular venous distention or bilateral pedal edema was appreciated. Initial electrocardiogram (EKG) on presentation showed typical atrial flutter with a rapid ventricular response (RVR), no acute ST-segment or T-wave abnormality (QRS:85 ms QTC:377 ms) (Figure 1). Initial laboratory values were significant for baseline normocytic anemia with hemoglobin of 10.2 g/dL (13.5-17.5 d/dL), acute kidney injury (AKI) on CKD stage five with creatinine (Cr) of 5.6 (0.6-1.3 mg/dL), estimated glomerular filtration rate (eGFR) of 12 mL/min/1.73 (>61 mL/min/1.73 m2), and slightly elevated liver function tests (LFTs), alkaline phosphatase (ALP) of 135 U/L (34-104 U/L), aspartate aminotransferase (AST) of 44 U/L (13-39 U/L), and alanine transaminase (ALT) of 43 U/L (7-52 U/L). Troponin was not elevated, thyroid stimulating hormone (TSH), and free T4 were within normal limits and were 1.471 (0.4-5.3 mc Intl Units/mL) and 1.1 (0.6-1.1 ng/dL), respectively. The urine drug screen was negative. Transthoracic 2D echocardiogram showed a left ventricular ejection fraction (LVEF) of 50% to 55%, mild left ventricular hypertrophy, moderately dilated left atrium(46 millimeters), mildly dilated right atrium, mild to moderate mitral valve regurgitation, pulmonary artery systolic pressure of 42.2 mmHg. Tricuspid valve and PA/RV systolic pressure - TR Max Velocity was 2.6 m/s.

His home medications included carvedilol 12.5 mg per oral (PO) twice a day (BID), hydralazine 50 mg PO three times a day (TID), clonidine 0.2 mg PO BID, and nifedipine 90 mg PO daily. He was noncompliant and did not take medications before presentation to the hospital. He was initially given home medications of carvedilol 12.5 mg BID and clonidine 0.2 mg PO BID along with an intravenous (IV) push of hydralazine 20 mg with no significant improvement in blood pressure. He was subsequently admitted to the cardiac critical care unit and nicardipine drip and hydralazine 50 mg TID were added to the antihypertensive regimen. Esmolol was avoided given the history of mild intermittent asthma. He remained persistently hypertensive despite initial management and in the setting of concomitant new-onset atrial flutter with rapid ventricular rate, nicardipine drip was switched to diltiazem drip. Diltiazem drip was up-titrated from 5 ml/hr to 10 ml/hr to 15 ml/hr overnight for better control of rapid ventricular rate. The next day, carvedilol was up-titrated to 25 mg BID; however, the patient remained persistently hypertensive. Nifedipine 90 mg orally daily was added to the antihypertensive regimen and diltiazem drip was switched to oral diltiazem 60 mg QID, nonsustained release form (diltiazem started after the carvedilol half-life is over (which is 7-10 hours). The patient started complaining of sudden onset, constant, diffuse non-radiating abdominal pain associated with one episode of non-bilious and non-bloody vomiting. The patient became hypotensive with mean arterial pressure (MAP) dropping below 60 mmHg, his heart rate acutely dropped to 40 bpm. The metabolic panel was significant for elevated lactic acid of 3.3 mmol/L (0.5-2.2 mmol/L), elevated lactate dehydrogenase (LDH) of 9646 U/L (140-271 U/L), markedly elevated LFTs with AST of 5,296 U/L (13-39 U/L) and ALT of 5,240 U/L (7-52 U/L), hyperkalemia with potassium of 7 (3.5-5 mEq/L), and AKI/acute tubular necrosis (ATN) with CKD stage five (Table 1).

**Table 1:** Trend of laboratory values during the hospital course CCB: calcium channel blocker; Cr: creatinine; eGFR: estimated glomerular filtration rate; AST: aspartate aminotransferase; ALT: alanine transaminase; LDH: lactate dehydrogenase

	On Admission	Post CCB Day 1	On Discharge	Reference Range
Cr	5.6	5.9>6.04>6.19>6.78	4.91	0.6-1.3 mg/dL
eGFR	12	10>9	13	>61 mL/min/1.73 m2
K	3.9	5.2>7	4.9	3.5-5 mEq/L
AST	44	764> 1925> 3845> 5296	49	13-39 U/L
ALT	43	495> 2043> 4928> 5240	625	7-52 U/L
Lactic Acid		3.3	1.4	0.5-2.2 mmol/L
LDH		572> 1440> 3837> 9646		140-271 U/L

Computerized tomography (CT) angiogram of the abdomen and pelvis was negative for mesenteric ischemia. Ultrasound of the abdomen did not show any evidence of cholelithiasis or acute cholecystitis. The antihypertensive regimen was discontinued, and resuscitation with fluids and Norepinephrine was started for cardiogenic shock management. The patient also started an Insulin drip with a 10% dextrose (D10) infusion. In addition, sodium zirconium cyclosilicate was administered for hyperkalemia. He became hemodynamically stable with a resolution of abdominal pain the next day. Norepinephrine, insulin, and D10 infusion were discontinued. During subsequent days, the patient was gradually transitioned back to home medications of carvedilol and clonidine and closely monitored in the telemetry unit, atrial flutter with RVR was spontaneously converted to normal sinus rhythm (the patient was on heparin drip for chemical anticoagulation), and he was later discharged with an adjustment in anti-hypertension (HTN) medications. The patient was counseled on medication compliance and avoiding further use of calcium channel blockers in the future. He followed up with the outpatient cardiology clinic.

## Discussion

The toxicity of calcium channel blockers (CCBs) disrupts cardiac inotropy, chronotropy, vascular smooth muscle contraction, and insulin secretion by directly inhibiting L-type calcium channels in the cardiac conduction system, myocardial and vascular smooth muscle cells, and pancreatic islet cells [5,6]. In addition, CCBs disrupt Ca-induced mitochondrial action and glucose breakdown, contributing to lactic acidosis [7]. At toxic doses, the tissue selectivity is overwhelmed, and the overall effects of toxicity cause hypotension, tissue ischemia, lactic acidosis, AV conduction abnormalities, and hyperglycemia [1,8]. Patients can rapidly deteriorate into refractory cardiogenic shock and distributive shock, acute respiratory distress syndrome (ARDS), end-organ injuries such as renal failure, myocardial infarction, ischemic bowel or pulseless electrical activity, and cardiac arrest [1,8]. Often, the diagnosis can be delayed if a patient does not present to the hospital with a known exposure. In our case, the patient was treated for hypertensive emergency and atrial flutter with non-dihydropyridines, dihydropyridines, and with other antihypertensives, in an effort to wean off the drips. The patient presented with a hypertensive emergency with concomitant atrial flutter with RVR and with underlying CKD and asthma; optimizing the medication regimen was challenging. The diagnosis was delayed due to the ambiguity of the symptoms at the time he started to deteriorate. It was ultimately the constellation of abdominal pain and bowel ischemia, hypotension, bradycardia, hyperkalemia from immense renal failure, and lactic acidosis that raised suspicion for CCB toxicity. However, it was ultimately a diagnosis of exclusion.

A retrospective analysis of 91 patients concluded that 38 cases of overdose were with verapamil, 31 cases were caused by nifedipine, and 24 cases by diltiazem ingestion [2]. There were fewer toxic manifestations with diltiazem ingestion compared to verapamil and nifedipine. However, of those who developed toxicities, conduction system abnormalities, and hypotension were the most commonly observed signs [2]. The mean toxic doses of verapamil and nifedipine were 3.2 grams and 340 milligrams, respectively [2]. Interestingly, our patient was dosed at appropriate doses but developed toxicity due to the overlapping effects of diltiazem, nicardipine, and nifedipine. Diltiazem was started after the carvedilol half-life was over (which is 7-10 hours). This is a unique cause of toxicity, as the majority of current literature describes cases of overdose with one CCB agent at a toxic dose. Only one other study examined the role of other antihypertensives and CCB overdose, in which case, combined overdoses of dihydropyridines and angiotensin enzyme inhibitors led to more severe hypotension, requiring significant hemodynamic support [9]. The timeline of switching between different CCB classes or to different medications such as beta-blockers can be better arranged for the overlapping effects.

The management of critically ill patients with CCB toxicity consists of supportive care to ensure airway protection, adequate breathing, and circulation [10]. Additionally, sufficient circulation can be established by intravenous fluids, vasopressors, inotropes, calcium, high-dose insulin, supplemental glucose, phosphodiesterase inhibitors, mechanical devices, such as pacemakers, and extracorporeal membrane oxygenation in patients with refractory shock [10]. In our patient, we did not have to resort to such mechanical device therapies [10]. The patient presented with a resistant hypertensive emergency, atrial flutter with RVR, and with underlying CKD; optimizing the medication regimen was challenging due to comorbidities. The lack of standardized guidelines warrants further studies to determine the best treatment for patients who overdose or have iatrogenic toxicity from CCBs [1,4].

## Conclusions

Intravenous CCBs are widely used for many clinical conditions, including abnormal heart rhythms and hypertensive emergencies. Due to its common usage in clinical practice, patients are susceptible to iatrogenic or accidental CCB toxicity. Toxicity causing end-organ damage can develop rapidly even within the therapeutic dose range. Especially in patients with concomitant entities, requiring to switch CCB classes can be challenging and requires further closer monitoring to avoid toxicity due to the lack of standardized protocol. Proper bridging of intravenous to oral CCBs is imperative to prevent the cumulative effect of the medication leading to CCB toxicity with cardiogenic shock.
